# A novel approach to enhance glioblastoma multiforme treatment efficacy: non-coding RNA targeted therapy and adjuvant approaches

**DOI:** 10.1186/s13148-025-01900-5

**Published:** 2025-06-21

**Authors:** Meijun Liu, Yuying Wang, Xiaoli Chen, Yu Zeng, Wenqiong Huang, Jiawen Yang, Hong Dai, Lixin Cheng, Claudio Mauro, Kenneth Chat Pan Cheung

**Affiliations:** 1 Phenome Research Center, Hong Kong Baptist University, Kowloon, Hong Kong China; 2 School of Life Science, Southern University of Science and Technology, Shenzhen, Guangdong Province China; 3 Health Data Science Center, Shenzhen People’s Hospital, First Affiliated Hospital of Southern, University of Science and Technology, Shenzhen, 518020 China; 4 Department of Chemistry, Hong Kong University of Science and Technology, Clear Water Bay, Kowloon, Hong Kong China; 5 College of Medicine and Health, University of Birmingham, Queen Elizabeth Hospital, Mindelsohn Way, Birmingham, B15 2WB UK

**Keywords:** Glioblastoma multiforme, Temozolomide, Disulfiram, Cuproptosis, Aspirin, Non-coding RNA

## Abstract

**Background:**

Glioblastoma multiforme (GBM) is a lethal brain tumor. With the current gold standard chemotherapy treatment, temozolomide (TMZ), many patients do not survive beyond one year. While the urgency of researching novel treatments is understandable, the prohibitively high costs and the prolonged duration of research and clinical trials significantly delay the availability of medical advancements to the general public. This highlights the urgent need for adjuvant therapies to enhance treatment effectiveness.

Main body: Recent research has suggested the potential of repurposing FDA-approved drugs such as temozolomide (TMZ), disulfiram (DSF), and aspirin for the treatment of glioblastoma, with encouraging evidence particularly for DSF and aspirin. Additionally, compounds like histone deacetylase inhibitors (e.g., vorinostat) are being investigated for their impact on non-coding RNA (ncRNA) modulation, including microRNAs (miRNAs) and long non-coding RNAs (lncRNAs). Combining traditional therapies with ncRNA modulation has shown potential in enhancing therapeutic efficacy and targeting specificity. NcRNAs play a crucial role in regulating gene expression and have been implicated in tumor growth, invasion, and treatment resistance. Recent discoveries, such as cuproptosis, offer new insights into tumor cell death mechanisms.

**Conclusion:**

This review focuses on how these molecular insights can serve as novel therapeutic targets and how drug adjuvant therapy may improve GBM treatment strategies. It focuses on how the integration of ncRNA modulation with conventional therapies and the combination strategy of enhancing efficacy of drugs can enhance treatment efficacy and pave the way for innovative approaches in managing GBM. In short, we will explore how non-coding RNAs (ncRNAs) might serve as promising targets and how repurposing TMZ, DSF, and aspirin could help enhance the efficacy of GBM treatment.

**Graphical abstract:**

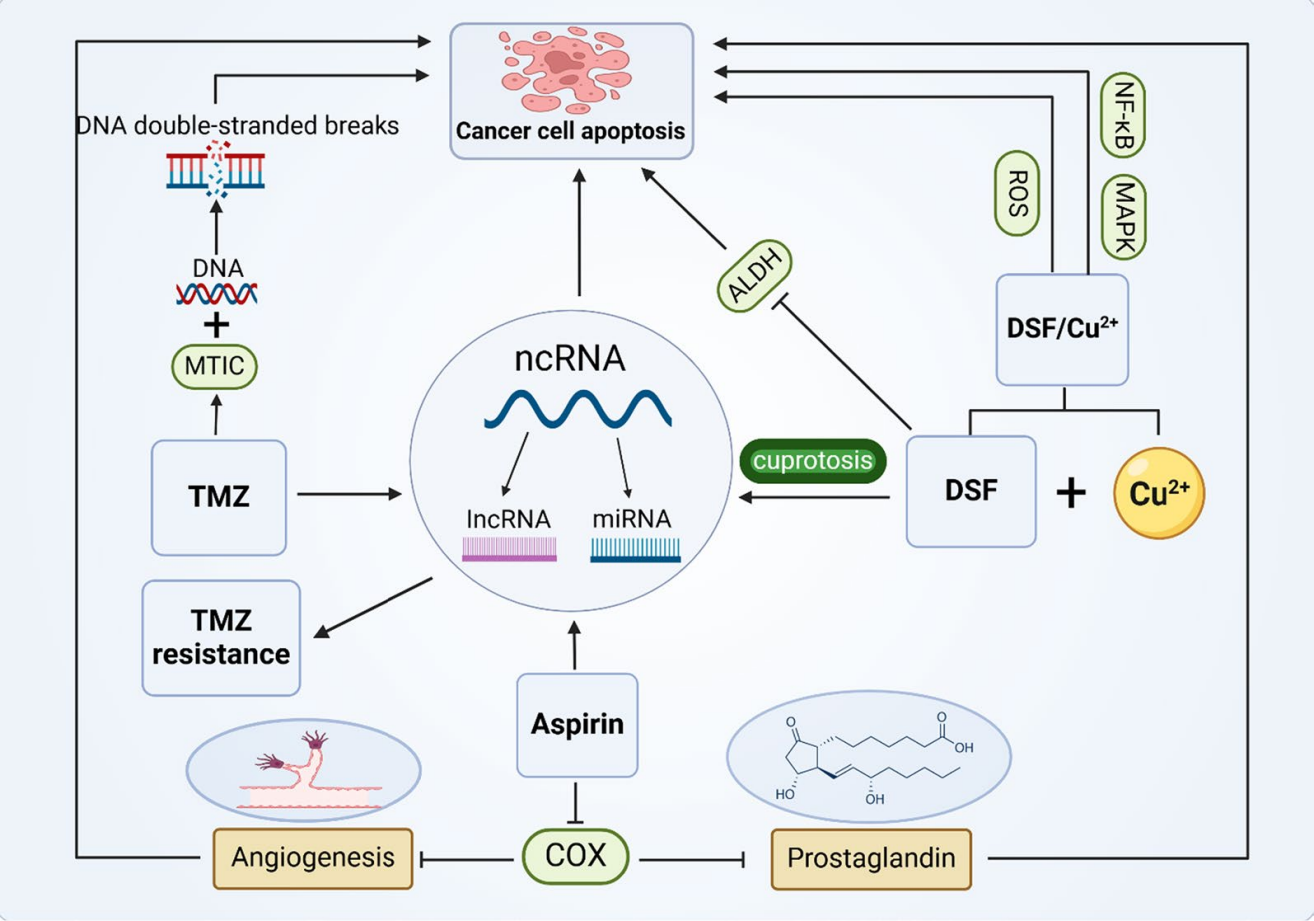

## Introduction

Glioblastoma multiforme (GBM) accounts for over half of all primary brain tumors, yet the detailed mechanisms underlying its origin remain uncertain even after decades of research [1].

Race, Sex, Age are the risk factors of GBM. In the case of US, being male, black, and increased age are important risk factors for GBM [2–4]. Researchers tried to characterize GBM into several subtypes based on GBMM gene expression profiles by RNA sequencing. Verhaak et al. identified four transcriptomic subtypes of GBMM: proneural, neural, classical, and mesenchymal, which were then validated in a separate 260 GBM cohort [5].8 Further studies using RNA-sequencing by Wang et al. have identified that only proneural, classical, and mesenchymal subtypes represent the glioma-intrinsic subtypes [6, 7]. NKAIN1 has the highest expression in the proneural subtype, while F13A1 has the highest expression in the mesenchymal subtype which is also associated with high infiltration of tumor-associated macrophages. Coagulation factor XIII A chain (F13A1) is a member of the blood coagulation cascade as a matrix cross-linker. Since extracellular matrix plays an important role in solid tumor growth, F13A1 may modulate aspects of the extracellular matrix to shape the tumor microenvironment in cooperation with tumor-associated macrophages [8].

The current hypothesis posits that proneural and classical GBM types may originate from neural stem cells and glial progenitor cells; however, this does not account for the onset of mesenchymal GBM [1]. Intriguingly, most GBM patients are unaware of the exact causes of their disease, although it represents the majority of malignant primary brain tumors. The only confirmed environmental risk factor is excessive radiation exposure [9]. According to a retrospective longitudinal study, symptoms exhibited by GBM patients include headache, nausea, vomiting, and papilledema, which can rapidly progress to cerebral herniation and death due to the aggressive nature of GBM [10].

Currently, the most common treatments for GBM patients remains surgical tumor resection, radiation oncology practice, and chemotherapy commonly with temozolomide (TMZ) [11]. Despite these interventions, the prognosis remains grim: many GBM patients succumb within one year of diagnosis, and only about 5% of patients are able to survive exceeding five years [1].

The standard treatment of glioblastoma comprises classical modalities surgery, radiotherapy, and chemotherapy with alkylating agent. However, molecularly targeted therapies and immunological approaches are currently undergoing clinical testing. Additional supportive care includes two prognostic biomarkers: mutations in isocitrate dehydrogenase (IDH) and O6-methylguanine-methyltransferase (MGMT) promoter methylation [12, 13]. These biomarkers assist in guiding therapeutic decisions and support efforts to personalize treatment approaches.

Researchers are actively exploring novel therapies, such as immune checkpoint inhibitors, effector immune cells, and oncolytic viruses. However, despite significant time and effort invested, breakthroughs yielding positive results have been limited [11]. Indeed, the estimated costs for the research and development (R&D) of a novel cancer-fighting drug can range from $944 million to $4.54 billion [14]. Furthermore, with many cancers being gene-specific, approximately 40% of the drugs designated to be labeled under 'orphan drugs'—which are aimed for treatments against rare diseases—target specific cancer subtypes. These drugs can carry staggering price tags, some exceeding $200,000 [15]. The prohibitively high costs of orphan drugs has prompted widespread calls for the development of more sustainable and affordable treatment options.

Importantly, the repurposing of existing drugs is a well-established strategy that offers the potential to treat diseases without incurring the exceedingly high costs associated with new drug research and development. Methods of drug repositioning (also known as drug repurposing) have been utilized to identify new therapeutic roles for existing drugs. Moreover, these repurposed drugs can often be marketed at reasonable prices. For example, sildenafil was initially invented and advertised as an antihypertensive medication, but Pfizer later rebranded it to treat erectile dysfunction. Similarly, thalidomide, originally a sedative, was banned by the government and withdrawn from the market, because it caused severe birth defects when taken by pregnant women. However, it was later discovered to be effective for other conditions, such as erythema nodosum leprosum and multiple myeloma [16]. Additionally, based on the principle of repurposing existing drugs for the treatment of GBM, multiple clinical trials are being conducted at the moment. Regorafenib, a multi-kinase inhibitor, and abemaciclib, an inhibitor of the CDK4/6-RB1 signaling pathway, are both accredited drugs that have previously been granted by Food and Drug Administration (FDA) for other indications. They are currently undergoing clinical trials to evaluate their potential anticancer efficacy against GBM [17].

This study aims to explore ongoing research efforts of enhancing FDA-approved drugs by adjuvant therapy especially focusing on the newly emerging field of non-coding RNAs (ncRNAs), being inspired by the successful example of diabetes mellitus and Alzheimer’s disease [18, 19].

Less than 10% of the human genome is transcribed, and only ~ 1% corresponds to protein-coding genes [20]. Other genomic regions are classified as non-coding RNAs. Emerging research revealed that ncRNA not only contributed to classification of GBM, but also have a crucial role in GBM pathogenesis [21], which then can affect the progression and expression of GBM. This leads to further insights into potential application of non-coding RNA species in glioma-grading and therapies. Long ncRNAs (lncRNAs), microRNAs (miRNAs), and circular RNAs (circRNAs) are all types of ncRNAs, which are commonly dysregulated in GBM. Dysregulation of ncRNAs can facilitate the invasion and metastasis of glioma [22].

In this review, we have chosen to primarily focus on TMZ, DSF, Aspirin and histone deacetylase inhibitors, all of which have been approved by FDA, which are the current medications due to their common characteristic of directly or indirectly targeting and influencing the promising non-coding RNAs (ncRNAs) expression and modulation. Their potential benefits are associated with GBM following their potential involvement to be adjuvant therapy.

## Methodologies

A comprehensive literature search was conducted using databases, such as PubMed, ScienceDirect and Google Scholar. The keywords employed included glioblastoma; temozolomide; disulfiram; cuproptosis; aspirin; non-coding RNA; histone deacetylase inhibitors. A Total of 250 articles published up to 2024 were retrieved. After applying inclusion criteria focused on the relevance of current treatment and non-coding RNA interactions in relation to GBM, 108 articles were selected for detailed analysis. The remaining articles were excluded due to a lack of relevance and insufficient evidence to explain the relationships associated with GBM pathogenesis and treatment effectiveness. The review includes clinical trials involving both humans and rats, also in vitro and in vivo experiments, including meta-analyses, systematic reviews, randomized controlled trials for discussion and evaluation. Successful clinical trials with positive outcomes shown toward other carcinomas are also included as potential option for GBM treatments.

### Epigenetic gene regulation and its role in cancer therapy

The structure of chromatin plays a crucial role in regulating gene expression through epigenetic mechanisms. These processes involve modifying DNA accessibility without altering its sequence, thus controlling gene expression. Key epigenetic mechanisms such as DNA methylation, histone modifications, and non-coding RNA activities influence this regulation. In cancer cells, like glioblastoma, epigenetic changes, especially in DNA methylation patterns, can have significant effects on the expression of important genes [23, 24].

Histone deacetylase inhibitors (HDACi) are a potentially beneficial group of compounds that block histone deacetylases, which are enzymes that remove acetyl groups from histone proteins. Through this inhibition, HDACi encourage a looser chromatin structure, aiding the transcription of genes that can hinder tumor growth and trigger cell death. Moreover, HDAC inhibitors have the ability to regulate non-coding RNAs, thus exerting additional control over gene expression and cellular functions.

An imbalance in histone deacetylase activity can impact regular cell growth, causing abnormal gene expression patterns and potentially playing a role in several diseases, such as cancer. When histone deacetylases' activity is not properly regulated, it can disturb normal cell growth, resulting in irregular gene expression patterns that are linked to various diseases, including cancer. Excessive activity or inhibition of HDACs can disrupt gene expression regulation, creating an environment favorable for uncontrolled cell division, compromised DNA repair, and resistance to cell death—common characteristics of cancer.

Significantly, elevated levels of HDAC8 have been detected in various tumors, notably neuroblastomas and gliomas. Studies have demonstrated that inhibiting HDAC8 in neuroblastoma cells can impede cell growth, decrease clonogenic potential, and lead to cell cycle arrest. HDAC inhibitors have the ability to trigger cell cycle arrest, promote cell differentiation and apoptosis, inhibit angiogenesis, and regulate immune responses. However, the impact of HDAC inhibitors may vary among different types of cancer [25–27].

At present, HDAC inhibitors are under investigation for a range of therapeutic applications, including neurodegenerative disorders, mood conditions, and cancer. In cancer treatment, these compounds are being studied for their ability to improve the effectiveness of traditional therapies and target the intricate molecular pathways involved in tumor development. In the case of glioblastoma, an aggressive brain tumor with poor outcomes, HDAC inhibitors have demonstrated potential in both laboratory and clinical research. They are thought to augment the impact of standard treatments like radiation and chemotherapy by altering the tumor microenvironment and encouraging the activation of tumor-suppressing genes [28].

GBM is the most prevalent and aggressive form of brain cancer in adults. Despite standard treatments—including surgical debulking, radiotherapy, and temozolomide chemotherapy—the median survival remains a mere 12–15 months. HDAC inhibitors have emerged as a novel class of cancer therapies, with several currently undergoing clinical trials for glioblastoma. The HDAC family consists of various classes, with most cancer-targeting inhibitors focusing on the zinc-dependent classes (I, II, and IV). Histone deacetylase inhibitors (HDACi) comprise structurally diverse compounds that are a group of targeted anticancer agents. One of the HDACi, vorinostat (suberoylanilide hydroxamic acid), has received FDA approval for treating patients with cutaneous T cell lymphoma [27]. With the successful example and the demonstration of the ncRNA modulation ability, this class of drugs can give the potential application to GBM treatment. By reprogramming the epigenetic landscape of glioblastoma cells, HDAC inhibitors may improve patient outcomes and offer a novel approach to combating this challenging malignancy.

Vorinostat, an HDAC inhibitor, has demonstrated promise in clinical trials, particularly for recurrent glioblastoma, and ongoing studies are exploring its use in combination therapies [29–31].

HDAC inhibitors demonstrate anticancer effects through various mechanisms, such as triggering cell cycle arrest and apoptosis, modifying gene expression to encourage differentiation, promoting oxidative stress, and displaying anti-angiogenic properties by reducing VEGF levels. Vorinostat and romidepsin have exhibited notable effectiveness against cancer cells, including those found in glioblastoma. Clinical trials in phases I and II have confirmed the safety and tolerability of vorinostat in different cancer types, with a phase II trial focusing on recurrent glioblastoma reporting a median survival of 5.7 months and a progression-free survival rate of 15.2% [32, 33].

Despite the encouraging findings, the actual clinical advantages of HDAC inhibitors such as vorinostat are still unclear and require additional exploration. Continued studies on the molecular subcategories of glioblastoma and the recognition of distinct epigenetic changes could aid in better patient selection and enhance treatment results with HDAC inhibitors. Due to their capacity to regulate ncRNA levels, HDAC inhibitors present a promising path for reshaping the epigenetic profile of glioblastoma cells, potentially improving patient outcomes and providing a new strategy to address this formidable cancer. For example, acetate-mediated HDAC inhibition in U87MG glioblastoma cells has been shown to increase the expression of several tumor-suppressive microRNAs involved in regulating proliferation, invasion, and angiogenesis [34].

### Treatment and epigenetics of GBM

GBM usually develops in the cerebral hemispheres. Due to its significant cellular heterogeneity, invasiveness, and regulation of the tumor microenvironment by glioma stem cells (GSCs), it is difficult to treat and relapse is almost inevitable [35]. The pathological characteristics of GBM include cell atypia, active mitosis, necrosis, and microvascular proliferation. Its heterogeneity and drug resistance are partly due to the complexity of different subtypes (classical, mesenchymal, and proneural), which show significant differences at the genetic and epigenetic levels, affecting prognosis and treatment response [5, 35]. GBM maneuvers the surrounding microenvironment to facilitate growth and evade immune attacks, by releasing immunosuppressive mediators (such as TGF-β, IL-10, and PGE-2). At the same time, GSCs alleviate T cell response by inducing PD-L1 [35–37]. Therefore, therapeutic strategies targeting these immune escape mechanisms may help improve patient survival, but single treatments are unlikely to be effective against such a heterogeneous cell population.

TMZ is the first choice for the treatment of primary GBM, especially in patients with MGMT promoter methylation. Although other treatments such as lomustine, bevacizumab and immune checkpoint inhibitors have shown different potentials, current research is still exploring their optimal use strategies and the effects of combination therapy [38].

The progression of GBM is closely associated with multiple epigenetic changes, including abnormal expression of microRNAs (miRNAs), circRNAs, and long non-coding RNAs (lncRNAs). miRNAs, such as increased miR-221 and reduced miR-128 and miR-181a/b/c [35, 39], affect apoptosis, proliferation, invasion, and chemoresistance of GBM [40, 41]. CircRNAs affect the biological behavior of GBM by regulating miRNAs and other mechanisms, and its stability makes it a potential biomarker [35, 42]. lncRNAs (such as HOTAIR, MALAT1, and SBF2-AS1) are crucial regulators of occurrence, progression, and drug resistance of GBM [43, 44]. Epigenetic drugs, such as HDAC inhibitors and PRMT5 inhibitors, are currently being tested in preclinical and clinical trials and have shown promise against GBM by modulating the expression of tumor-related genes and cellular processes [45, 46].

### Non-coding RNA (ncRNA) in anticancer research of glioblastoma

Non-coding RNAs (ncRNAs) can be distinguished based on their size (measured in nucleotides) into two main categories: short regulatory ncRNAs and long non-coding RNAs (lncRNAs). Short regulatory ncRNAs are further categorized into four subgroups based on their structure and function: microRNAs (miRNAs), short interfering RNAs (siRNAs), Piwi-interacting RNAs (piRNAs), and small nucleolar RNAs (snoRNAs). Although ncRNAs do not typically participate in protein synthesis directly, they may still impact on cancer progression, malignancy, invasion, and angiogenesis [47, 48].

In the broader context of cancer, alterations in ncRNA regulatory networks have been linked to either oncogenic or antitumor effects. Specifically in GBM, researchers have identified over 250 upregulated and nearly 100 downregulated miRNAs as statistically significant, offering potential as biomarkers or therapeutic targets. Among these, miRNA Mir-21 has been extensively studied and was the first to be recognized as an oncogenic miRNA. Its suppression can trigger anti-apoptotic and pro-survival pathways. miR-10b is specifically expressed in glioma tumors, correlating with malignancy grade and promoting invasion by repressing HOXD10 and modulating MMP14 and uPAR. Besides, MiR-221/222 is also overexpressed in GBM, enhancing cell proliferation and migration while inhibiting apoptosis.

Similarly, long non-coding RNAs (lncRNAs) have been observed to be abnormally expressed in GBM, with various lncRNAs either promoting or inhibiting tumor growth. LncRNAs, such as miR-199a, act as a molecular sponge for miRNAs, promote cell proliferation and tumorigenesis, and lead to resistance to TMZ-treatment. While MEG3 and HOTAIR have been implicated in regulating key oncogenic pathways, other lncRNAs like CASC2 and RAMP2-AS1 have tumor-suppressive roles, with CASC2 enhancing chemosensitivity through autophagy inhibition [48]. In addition, exosomal ncRNAs, including circular RNAs, microRNAs, and long non-coding RNAs, can regulate drug resistance in GBM through various signaling pathways or by modulating regulatory proteins and their corresponding genes [49].

Leveraging insights into ncRNA interactions, traditional medications such as TMZ, DSF, and Aspirin can be repurposed for the treatment of GBM. This approach requires optimizing their efficacy and overcoming potential drug resistance issues. Future studies could focus on validating ncRNA biomarkers in clinical trials and exploring the combination of ncRNA-targeted therapies with traditional treatments to enhance efficacy and reduce side effects.”

### Enhancing the efficacy of temozolomide to treat glioblastoma by targeting ncRNA

Temozolomide (TMZ) has been regarded as the cornerstone chemotherapeutic agent for treating GBM, typically administered in conjunction with radiotherapy. As an oral alkylating agent, its primary mechanism of action involves inflicting DNA damage to induce apoptosis of GBM cells [50].

A meta-analysis has provided robust evidence that the addition of adjuvant TMZ to radiation therapy can significantly improve patient survival, demonstrating a 37% reduction in the hazard rate (95% CI 0.24–0.48; *P* < 0.00001) compared to a control group receiving only radiotherapy [51].

The methyl diazonium ion (CH_3_N_2_+), generated from TMZ, serves as the principal alkylating species, capable of methylating DNA at various sites including N7 of guanine (N7-G), N3 of adenine (N3-A), and O6 of guanine (O6-G). Methylation at N7-G and N3-A is typically reversible through the base excision repair (BER) pathway. However, methylation at the O6 position of guanine, resulting in O6-methylguanosine (O6-MeG), plays a pivotal role in TMZ's antitumor efficacy. This modification instigates a series of futile mismatch repair (MMR) cycles, leading to impediments of replication forks, subsequently DNA double-strand breaks and eventually apoptosis triggered by extensive DNA damage [50, 52].

Resistance to TMZ can arise due to the DNA repair activity of O6-methylguanine DNA methyltransferase (MGMT), which specifically reverses methylation at the O6 position of guanine. Overexpression of MGMT or defects in the MMR system are known to contribute to this resistance [50]. Recent studies have identified several ncRNAs that have the potential to inhibit MGMT expression, thereby mitigating TMZ resistance.

Although the overexpression of the lncRNA FOXD2-AS1(Bgee database: mostly expressed in mucosa of transverse colon, descending thoracic aorta and popliteal artery in humans) has been previously associated with tumor progression in GBM, recent studies have demonstrated that knockdown of FOXD2-AS1 can attenuate TMZ resistance in human glioma cell lines U251 and A172. This is achieved by reducing the methylation levels of the MGMT gene, suggesting that FOXD2-AS1 might be a promising therapeutic target to investigate for raising TMZ efficacy in GBM treatment [53, 54].

The lncRNA Urothelial Cancer Associated 1 (UCA1) has been implicated in the proliferation of bladder cancer cells by modulating various genes (Bgee database: mostly expressed in endometrium). Notably, both MGMT and lncRNA UCA1 expression levels have been found to be elevated and strongly correlated in GBM tissues and cell lines. Recent research has unveiled that the lncRNA UCA1/miR-182-5p/MGMT regulatory axis can play a pivotal role in reducing TMZ resistance in a total of 17 glioma tissues (9 World Health Organization [WHO] grade III and 8 WHO grade IV), 6 noncancerous peritumoral brain edema tissues (adjacent tissues) originated from patients in Sichuan Provincial People’s Hospital from July 2017 to July 2019 and BALB/c nude mice (Changsha SLAC Laboratory Animal Company, Changsha, China). Silencing lncRNA UCA1 through siRNA can lead to the knockdown of MGMT-related DNA repair pathways. Specifically, miR-182-5p has been shown to suppress MGMT expression by targeting both lncRNA UCA1 and MGMT directly. Overexpression of miR-182-5p has been associated with the reversal of TMZ resistance in GBM, offering a potential therapeutic strategy to overcome drug resistance [54, 55].

The lncRNA termed lnc-TALC (temozolomide-associated lncRNA in GBM recurrence) has been identified as a critical factor in the resistance to TMZ and the recurrence of GBM in patients. lnc-TALC(Bgee database: expressed most in testis, sperm and bone marrow) promotes TMZ resistance from competitively binding to miR-20b-3p, in order to facilitate the expression of c-Met and concurrently upregulate MGMT expression in human GBM LN229 and U251 cells, patient-derived GBMM cells 551 W and HG7 and their TMZ-resistant derivatives. Based on clinical survival curves from 79 GBM patients from The Second Affiliated Hospital of Harbin Medical University, intriguingly, although GBM patients with low levels of lnc-TALC receive better responses by treatment with TMZ, elevated lnc-TALC patients who undergo TMZ treatment have been observed to have worse outcomes and shorter survival times compared to those who do not receive TMZ therapy. This underscores a complex role of lnc-TALC in diminishing the anticancer efficacy of TMZ in GBM treatment [54, 56]. This paradoxical observation warrants further research to elucidate the precise role of lnc-TALC in GBM pathophysiology and its impact on treatment outcomes.

Initially calculating from Alzheimer's Disease Dataset (Salomon (GSE5281) [57], Cotman (GSE48350) [58], GTEx [59] and ICGC GBM-US [60]), GBM stem cell models, G7 and G26, the lncRNA TP73-AS1(Bgee database: expressed most in ovary, uterus and saphenous vein) is responsible to TMZ resistance as well as to mechanisms of brain aging. TP73-AS1 is known to exert its effects through the interaction with Yin Yang 1 (YY1), a transcription factor that regulates tumorigenesis and the cellular response to chemotherapy [54, 61].

Additionally, the suppression of another lncRNA ZBED3-AS1 has been observed in U87-MG cell and G131212 GBM cell TMZ-resistant derived cells. This downregulation of ZBED3-AS1(Bgee database: mostly expressed in germ, corpus callosum and calcaneal tendon) leads to the activation of Thrombomodulin (THBD), which is associated with enhanced resistance to TMZ. Moreover, microRNA miR-519a(Bgee database: most microRNA 519a-1 expressed in adrenal gland, stomach and esophagus mucosa; most microRNA 519a-2 expressed in heart left ventricle, right ovary and blood) can induce programmed cell deaths in GBM cells, enhance the sensitivity of these cells to TMZ, and promote autophagy by targeting the STAT3/Bcl-2/Beclin-1 pathway [54, 62]. Similarly, miR-182 (Bgee database: mostly expressed monocyte, pancreas and blood) holds a multifaceted role in monitoring GBM tumor: a promoter of differentiation in GBM cell lines, an inhibitor of GBM cell proliferation, and a sensitizer of GBM cells to TMZ-induced apoptosis, based on analysis on the dataset from Cancer Genome Atlas program [54, 63].

Collectively, these studies on ncRNAs not only illuminate complex mechanisms that contribute to GBM resistance to TMZ but also suggest promising therapeutic strategies. By integrating ncRNAs such as miR-519a and miR-182 with the current standard-of-care chemotherapy, TMZ, there is the potential to both diminish resistance and enhance chemotherapeutic efficacy. These preliminary results underscore the essence of ongoing research to fully understand the functions and impacts of ncRNAs in GBM treatment, with the aim of developing more effective combined therapeutic regimens.

In addition to ongoing efforts to enhance the efficacy of TMZ by the newly available ncRNA, other adjuvant therapy attempts have been also intensively investigated. Due to the paper limits and scope of this, we will only introduce a few TMZ adjuvant therapy trials that have been tested in clinical trial phase III: addition of immune checkpoint inhibitor nivolumab to the standard-of-care radiotherapy+ TMZ resulted in a three-month decrease in overall survival and a higher frequency of severe adverse events in a single-blind randomized clinical phase III trial containing 716 patients newly diagnosed with WHO grade IV malignant glioma with methylated MGMT promoter [64]. However, interferon alfa adjuvant was found effective to extend patients overall survival from an average 18.8 [95% CI, 16.9–20.7] months to 26.7 [95% CI, 21.6–31.7] months based on 199 enrolled patients across 15 Chinese medical centers [65].

### Repurposing disulfiram (DSF) to treat GBM by targeting ncRNAs through cuproptosis

#### DSF and its antitumor effect against GBM

**Fig. 1:**
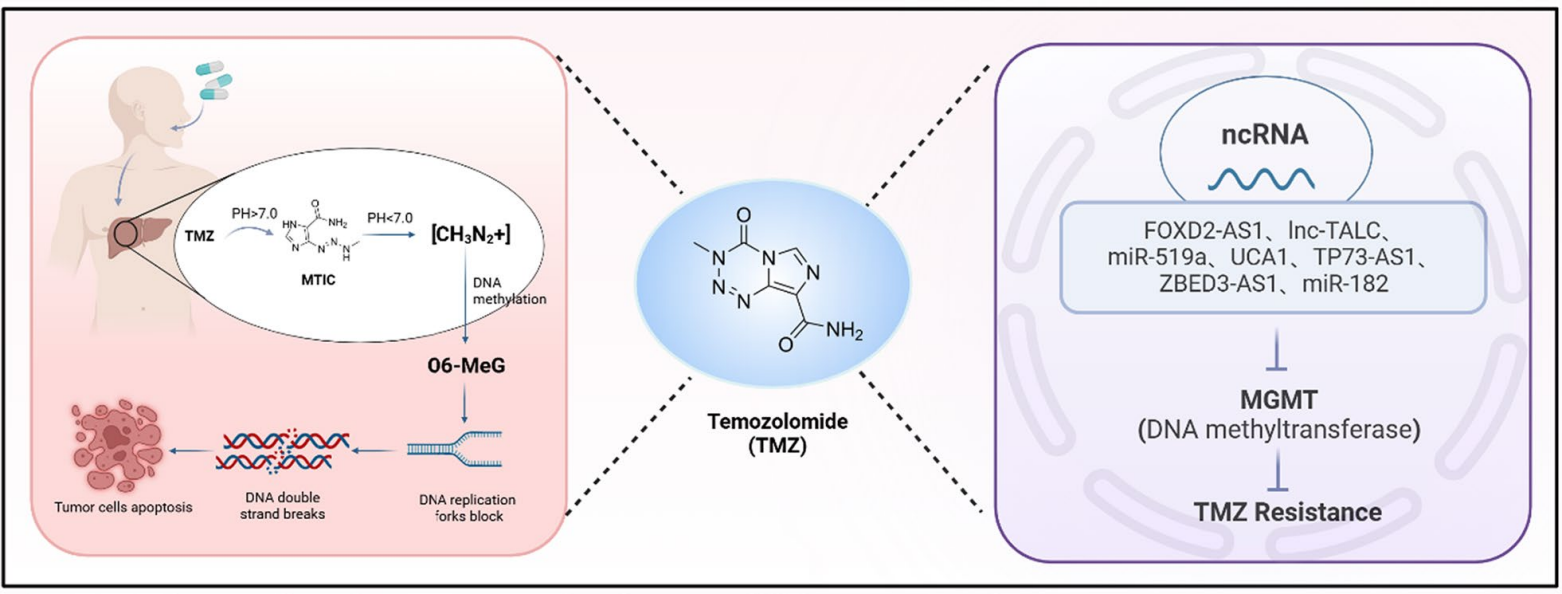
The chemical structure and detailed mechanism of the medical effect of TMZ. The left part shows that temozolomide is metabolized to its active form MTIC by esterase and carbonic anhydrase. MTIC causes DNA damage by double strand breaks to tumor cells and induces apoptosis. The right part shows that MTIC binds to DNA through an additional reaction, resulting in DNA double-strand breaks. The antitumor activity of TMZ begins with the hydrolysis of its tetrazine ring under basic conditions. This reaction transforms TMZ into the active intermediate 3-methyl-(triazen-1-yl) imidazole-4-carboxamide (MTIC), accompanied by the release of H_2_O and CO_2_. Under acidic conditions, MTIC further degrades into 5-aminoimidazole-4-carboxamide (AIC) and a highly reactive methyl diazonium ion (CH3N2+) [45, 47]

Disulfiram (DSF) was first approved by the FDA in 1951 and has since been used to treat alcoholism without any records of intolerable side effects. As a safe and well-documented drug, it is also recognized as a highly effective inhibitor of aldehyde dehydrogenase (ALDH), capable of downregulating all known cytosolic and mitochondrial ALDH isoforms. Especially ALDH1A3, a member of the ALDH family, is involved in the metabolism of *all-trans-*retinoic acid (*at*RA); overexpression of ALDH1A3 in brain and overproduction of *at*RA have been associated with proliferation of GBM cancer cells and worsened patient survival (Fig. 1). On the contrary, severe GBM patients have higher chances of surviving more than 3 years if they express low levels of ALDHA1 and ALDH1A3 [66].

Furthermore, copper-dependent cytotoxicity and consequential cell deaths are more dominant in cells that utilize oxidative phosphorylation as their energy sources [67]. Targeting oxidative phosphorylation to inhibit excessive ATP production and reduce reactive oxygen species have been reasonable strategies against GBM [68, 69].

**Fig. 2:**
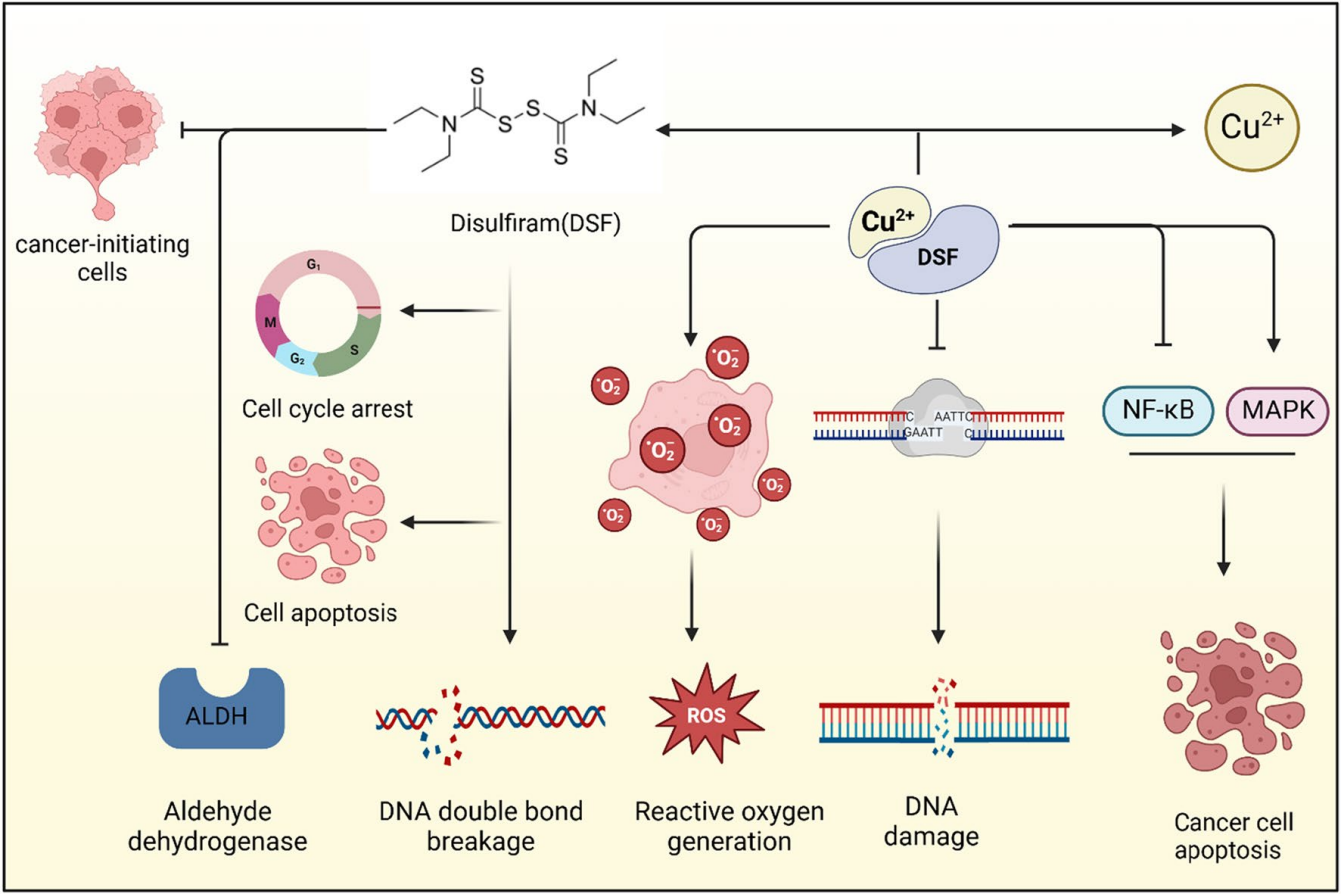
Antitumor mechanisms of Disulfiram/Copper adjuvant therapy. Disulfiram acts on cancer-initiating cells by inhibiting ALDH, preventing their growth and leading to cell cycle arrest and cell apoptosis. At the same time, the copper-disulfiram complex (DSF/Cu^2^⁺) induces the generation of ROS, destroys the DNA structure, and causes DNA damage and DNA double-strand breaks. In addition, the complex further enhances the apoptosis of tumor cells by regulating the NF-κB and MAPK signaling pathways

DSF has captured the interest of researchers in exploring its antitumor potential against cancer by inhibiting ALDH and inducing copper-related cell deaths. This has led to investigations into how DSF might serve as a novel therapeutic target, a topic that has been under study for decades, as illustrated in Fig. 2 [70]. Disulfiram acts on cancer-initiating cells by inhibiting ALDH, preventing their growth and leading to cell cycle arrest and cell apoptosis. At the same time, the copper-disulfiram complex (DSF/Cu^2^⁺) induces the generation of ROS, destroys the DNA structure, and causes DNA damage and DNA double-strand breaks. In addition, the complex further enhances the apoptosis of tumor cells by regulating the NF-κB and MAPK signaling pathways.

The initial documented application of DSF for cancer treatment was in 1961, when Dr. Lewison of Johns Hopkins Hospital administered DSF to a patient with breast cancer. This treatment led to the complete eradication of metastases in the spine, ribs, and pelvis, and there was no recurrence for the subsequent decade, from 1961 to 1971. In a later double-blind trial, the survival rate for patients treated with DSF was observed to be 81%, a significant improvement compared to the 55% survival rate in the control group. Nevertheless, the effectiveness of DSF as a sole antitumor agent is compromised by its short half-life. Researchers soon found that the addition of copper to DSF might improve its efficacy. To be more specific, DSF/Cu therapy typically suppresses DNA repair pathways in tumor cells and induces redox reactions and reactive oxygen species (ROS) generation. This inhibition of the proteasome results in an increased vulnerability to DNA damage, leading to apoptosis of tumor cells [70].

#### Successes of DSF and DSF/Cu therapy against cancers and GBM experimentally

Indeed, plentiful of in vitro and in vivo experiments have shown promising results regarding DSF and DSF/Cu^2+^ treatments against cancer and GBM. In human pancreatic cancer cell lines PANC-1 and SW1990, both independent treatment of DSF and combination treatment DSF/Cu^2+^ showed antitumor effects and increases the susceptibility to radiotherapy, resulting in cell cycle arrest, apoptosis as well as radiation-induced DNA double-strand breaks (DSBs) [71]. Similarly, treatment with only DSF can enhance the effectiveness of cisplatin-induced apoptosis in primary lung adenocarcinoma by downregulating ALDH1A3 and targeting cancer-initiating cells (CICs) which originated from patient surgical resection samples at City of Hope, one at Ib stage with mutated p53 and KRAS, one at Ib stage without DNA testing and one at Ia stage also without DNA testing [72]. The combination of DSF with copper further amplifies the inhibitory effects on ALDH in stem-like ovarian cancer cell lines IGROV1, SKOV3 and SKOV3IP1 with ALDH + phenotype, outperforming the effects of DSF alone, copper alone, or the specific ALDH inhibitor diethylaminobenzaldehyde (DEAB). This combination also increases the generation of reactive oxygen species (ROS) [73].

In the specific context of GBM, in U251MG, U87MG and U373MG GBM cell lines, the DSF/Cu combination has successfully induced apoptosis and inhibited the proliferation of ALDH-positive neurosphere cells, potentially by upregulating the MAPK pathway and downregulating NFκB activity [74]. Similarly, combining a specially designed DSF-loaded, ion-sensitive nanoemulsion in situ gel (DSF-INEG) with copper (forming DSF-INEG/Cu) has successfully inhibited the growth of GBM tumor cells, specifically C6 and U87, in vitro. Both the nose-to-brain delivery of DSF-INEG/Cu and the standard DSF/Cu treatment have significantly prolonged the survival time of mice in vivo [75].

#### Failure of DSF and DSF/Cu in clinical trials against GBM

However, despite multiple successes of experimental studies of DSF and DSF/Cu^2+^ therapies in the labs, their efficiency failed to translate well into real clinical benefits. In a phase I clinical trial, which was led by Dr. Huang J, containing 18 GBM patients, both DSF and DSF/Cu^2+^ showed indistinguishable effects with each other, showing no obvious clinical benefits [76]. In the subsequent open-label, single-arm phase II study, led by same first author Dr. Huang, involving 21 recurrent TMZ-resistant GBM patients, only 14% experienced clinical benefits and overall response rate was zero [77]. According to Dr. Huang’s most recent publication, phase I/II clinical trial of 33 GBM patients which included 12 IDH-mutant, 9 NF1-mutant, 3 BRAF-mutant, and 9 other IDH-wild-type cases and over two years of median follow-up time, addition DSF/Cu to current TMZ therapy did not show a statistically significant improvement in outcomes for GBM patients compared to standard TMZ therapy; though extended remissions were observed in three patients with BRAF mutations [78].

The null results were further validated by a randomized clinical trial involving 88 patients with recurrent GBM, recruited from 7 study sites in Sweden and 2 sites in Norway from 2017 to 2020 treated with DSF or DSF/Cu^2+^. Despite patients were merely suffering from more frequent severe adverse reactions in DSF/Cu^2+^ treatment group (41% in DSF/Cu^2+^ treatment group vs 16% for DSF treatment group), they also had shorter median survival time (5.5 months in DSF/Cu^2+^ treatment group vs 8.2 in DSF treatment group) [79].

Thus, it seems relatively obvious that either DSF alone or DSF/Cu^2+^ combination treatment might be arduous to alleviate GBM in most patients. Although hopes might still be held up considering that DSF alone or DSF/Cu^2+^ combination treatment would be effective against specific mutants of GBM cancer in certain subtype of patients, additional adjustments are needed for DSF and DSF/Cu^2+^ combination treatment to better execute their antitumor effects clinically in real patients.

#### Possibility of improving efficacy of DSF/Cu by utilizing ncRNA

Recently, the copper-induced cell death, cuproptosis was recognized as a distinctive form of cell death [80]. A recent genomic analysis has already indicated that gene ATP7A, which is a copper transporter contributing to radiotherapy resistance in cancer, was highly associated with most of other 16 cuproptosis genes and loss of ATP7A can disrupt transportation of copper and reinforce cuproptosis, which might serve as a promising target for DSF and DSF/Cu2+ combination treatment [81–84]. ATP7A is widely expressed in heart, brain, lung, muscle, kidney, pancreas, and to a lesser extent placenta [85, 86] and thus additional engineering or special delivery strategy will be needed to improve specificity and avoid potential off-target effects in other organs when considering to apply to humans. Knockdown of ATP7B (Bgee database: mostly in human nose, testis and liver) using siRNA was also found to strengthen the antitumor effects of DSF/Cu2+ in docetaxel-resistant prostate cancer cell lines: DU145-docetaxel resistant (DR), 22Rv1-DR, LNCaP-DR, and PC3-DR cell lines by downregulating expression of COMMD1 and p21/WAF1 genes [54, 87].

Another genomic analysis has revealed that miR-606 can inhibit the glycolysis and proliferation of GBM cells by binding to and regulating the mRNA of the cuproptosis-related gene ferredoxin 1 (FDX1), regulation of which might also improve the efficacy of DSF and DSF/Cu2+ combination treatment [88]. Similar to ATP7A, miR-606 is mostly expressed in kidney, myometrium and liver, according to Bgee database and a concern of lack of specificity of rudimentary form of miR-606 arises, resulting in immature clinical applications [54].

Some ongoing experiments have alluded to the possibility of enhancing DSF or DSF/Cu2+ treatment by combining with RNAs. Atg5 siRNA (Bgee data base: mostly expressed in primordial germ cell in gonad, colonic mucosa and mucosa of sigmoid colon) could suppress autophagy and augment DSF/Cu-induced apoptosis in human non-small cell lung cancer (NSCLC) cell lines (A549, NCI-H460 and NCI-H1299) and in xenograft mice models [54, 89]. Disulfiram and NPL4(Bgee database: existing in humans as homology NPLOC4 which expressed mostly in gastrocnemius, apex of heart hindlimb stylopod muscle) siRNA successfully inhibited human renal cell carcinoma (RCC) cell lines 786-o, A498, Caki1, and Caki2 and in xenograft models by potentially quelling serine biosynthesis and aldose reductase-related genes [54, 90].

Beyond GBM, additional genomic analyses have investigated the relationship between cuproptosis, cuproptosis-related lncRNAs, and various other cancers. Utilizing transcriptome expression data and validation in HepG2 and MHCC-97H hepatocellular carcinoma cell lines, AC099329.2 (Bgee database: a novel gene, highest expressed in liver, reproductive and placenta)and DNMBP-AS1(Bgee database: mostly expressed in colon, nose and none marrow) have been identified as having potential protective effects, while AC138904.1(Bgee database: heart, testis, skeleton muscle), DEPDC1-AS1(Bgee database: ganglionic eminence, bone marrow, leg muscle), GIHCG(Bgee database: testis, adenohypophysis, leg muscle), and AC145343.1(Bgee database: testis, buccal mucosa cell, gonad) may be oncogenic [54, 91]. In the case of lung adenocarcinoma (LUAD), 16 cuproptosis-related lncRNAs have been found to correlate strongly with the condition in patients and may serve as suitable biomarkers for the disease, based on recent statistical analysis of genomic and clinical data: LINC00592(Bgee database: testis, buccal mucosa cell, amniotic fluid), ZNF571-AS1(Bgee database: testis, cortical plate and colonic epithelium), and SEPSECS-AS1(Bgee database: buccal mucosa cell, testis and heart) were tested in survival analysis in GSE30219, GSE31210, GSE37745 Gene Expression Omnibus (GEO) datasets. High levels of LINC00592 showed strong statistical significant correlation in reducing patient survival rates in GSE30219 (p=0.001), but absent in other two datasets, while high levels of SEPSECS-AS1 exhibited strong protective effects (*p *< 0.001) in terms of patient survival in GSE31210 but also nonexistent in remaining two datasets [54, 92]. Despite being mere biomarkers, overexpression of the long non-coding RNA cancer susceptibility candidate 8 (lncRNA CASC8, Bgee database: olfactory segment of nasal mucosa, ganglionic eminence, minor salivary gland) has been observed in pancreatic cancer (PC). Inhibition of lncRNA CASC8 reduces cell proliferation, impedes PC cell migration, and promotes apoptosis in tumor cells [54, 93].

As RNA research further advances and knowledge of cuproptosis develops, more GBM-specific and cuproptosis-related lncRNAs will likely be identified and further investigated; thus, clinical efficacy involving DSF and DSF/Cu would be further elevated.

#### Aspirin and its antitumor effects against GBM

The identification of the chemical formulation of aspirin can be traced back to Dr. Felix Hoffman in 1897. Aspirin is a widely used traditional drug with a well-proven antithrombotic effect for the secondary prevention of cardiovascular disease and a more controversial role in primary prevention [94].

**Fig. 3:**
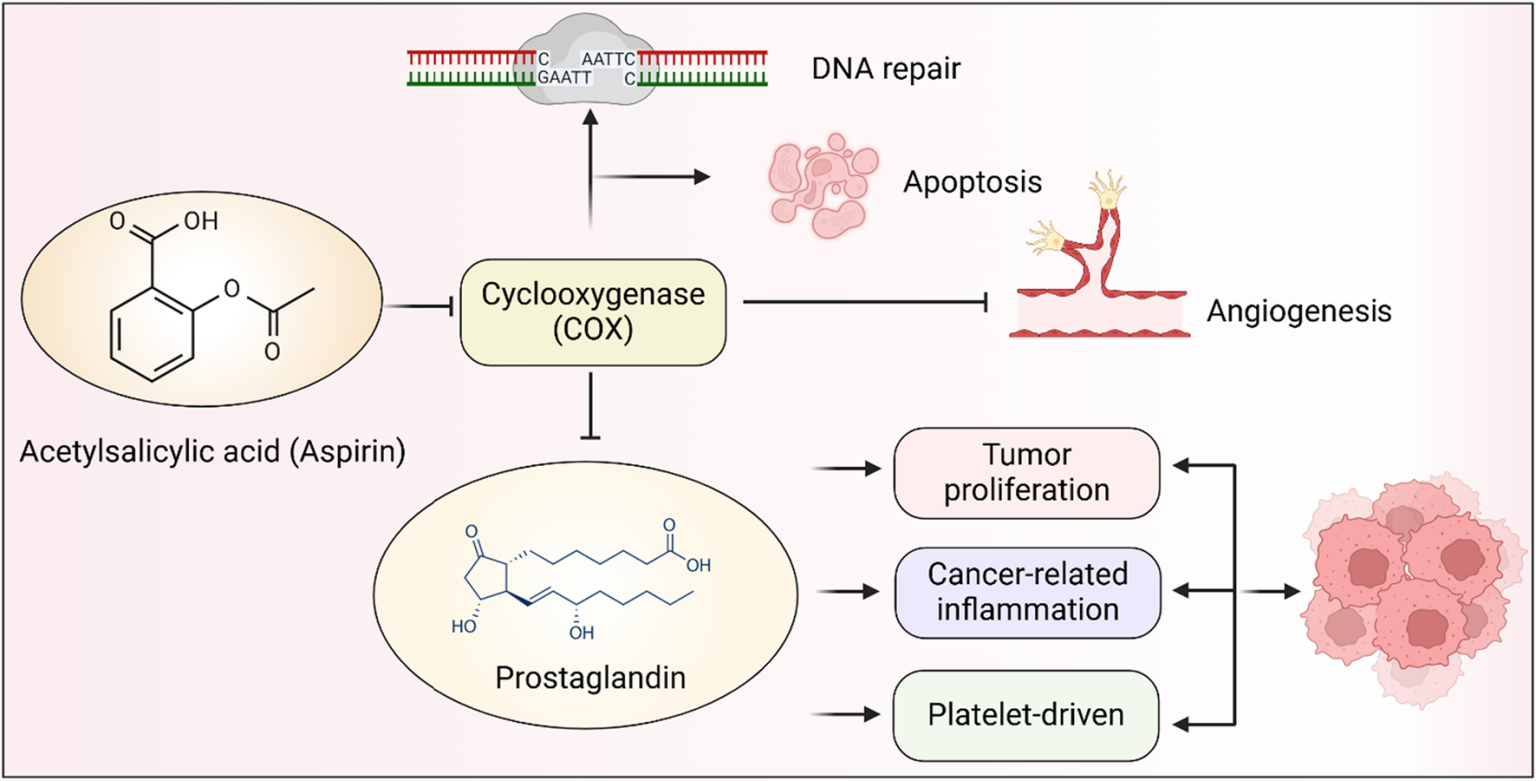
Antitumor mechanisms of Aspirin. Aspirin inhibits the production of prostaglandins by blocking COX activity, thereby affecting various biological processes related to tumor progression. Specifically, by reducing the synthesis of prostaglandins, aspirin effectively inhibits tumor cell proliferation, cancer-related inflammation, and platelet-driven carcinogenesis. In addition, aspirin promotes DNA repair and induces tumor cell apoptosis, while inhibiting angiogenesis, further preventing tumor growth and spread

Researchers have uncovered several mechanisms by which aspirin might intervene in or even directly treat cancer. The primary mechanism may be the aspirin's ability to disrupt essential cancer signaling pathways by targeting signaling molecules known as prostaglandins through the downregulation of the cyclooxygenase (COX) enzyme. By causing this disruption, aspirin can inhibit tumor growth by impeding proliferative pathways, reducing cancer-associated inflammation, and diminishing platelet-driven pro-carcinogenic activity. Other mechanisms include the suppression of angiogenesis through COX enzyme inhibition, the promotion of apoptosis, and the stimulation of p53-induced DNA repair, as illustrated in Fig. 3 [95]. Aspirin inhibits the production of prostaglandins by blocking COX activity, thereby affecting various biological processes related to tumor progression. Specifically, by reducing the synthesis of prostaglandins, aspirin effectively inhibits tumor cell proliferation, cancer-related inflammation, and platelet-driven carcinogenesis. In addition, aspirin promotes DNA repair and induces tumor cell apoptosis, while inhibiting angiogenesis, further preventing tumor growth and spread.

In a recent bioinformatic study using Boolean network and known pathways active in GBM, researchers found that aspirin appears to work well against most GBM cancer cell lines and seems to be able to reduce TMZ resistance when used in combination with TMZ. More intriguingly, combinations of aspirin with palbociclib and of aspirin with AT7519 have been identified as the best combination therapy candidates, which require further in vitro and in vivo experimental confirmation [96]. Regardless, the computer simulation study highlights the previously overlooked antitumor potential of aspirin against GBM.

Indeed, aspirin has been found to downregulate the proliferation biomarkers c-myc, cyclin D1, and fra-1 mRNA and protein in glioma cells lines: U87 and A172 by suppressing the β-catenin/TCF signaling pathway [97]. A combination of aspirin and the nitric oxide (NO) producer JS-K can induce necrotic cell death through apoptosis and the disruption of calcium levels, facilitated by COX inhibition, in glioma cell lines U87MG and LN229 [98]. Testing in T98G human GBM cells, another combination therapy of aspirin and indomethacin has successfully induced apoptosis and inhibited the proliferation of tumor cells [99]. Aspirin combination therapy with TMZ, bevacizumab, and sunitinib has also inhibited tumor formation by blocking angiogenesis and increasing cell sensitivity in human primary GBMM-endothelial cells which originated from five GBM patients after surgical resection at the Neurosurgery Unit of Fondazione IRCCS Ca’ Granda Ospedale Maggiore Policlinico (Milan, Italy) [100]. In LN229 and U87 GBM cell lines, aspirin/TMZ microspheres have been shown to have significant better antitumor effects compared to aspirin-only and TMZ-only treatment groups. The improvement in the aspirin/TMZ combination therapy can be characterized by significantly higher apoptosis rates, fewer tumor colony formations, almost complete inhibition of tumor growth, or even moderate shrinkage of tumor size [101].

Additionally, in a case–control study, nonsteroidal anti-inflammatory drugs (NSAIDs) have been found to significantly reduce the odds ratio in GBM patients, with aspirin particularly able to reduce the odds ratio by nearly 50%, based on 236 incident GBM cases and 401 population-based controls from the San Francisco Bay Area Adult Glioma Study [102]. However, another case–control study partially contradicted the risk-reducing effects of aspirin by only having a statistically insignificant average odd ratio of 0.9 in 2688 glioma cases and 18 848 population controls recruited in Denmark [103]. A recent glioma international case–control study and a meta-analysis published in 2020 using patients data from Glioma International Case-Control Study (GICC) which included 4,533 glioma cases and 4,171 controls during 2010–2013 supported a roughly 40% hazard reduction rate by daily use of aspirin and a 16% marginally statistically significant prevention effects of aspirin against GBM by the meta-analysis [104]. Overall, aspirin is expected to have a moderate to weak effect against GBM, which requires further investigation to warrant and optimize its effectiveness.

Overall, the existence of aspirin's antitumor potency against GBM seems to be generally agreed upon by different studies, but whether the antitumor effects of aspirin hold up clinical significance and justify the additional financial costs and adverse effects remains to be further investigated. We propose that ncRNAs might optimize aspirin to be more effective against GBM.

#### Repurposing aspirin together with ncRNAs

Unfortunately, to the best of our knowledge, there are no previous studies that have directly attempted to utilize aspirin and ncRNAs to treat GBM. However, there is abundant research on repurposing aspirin with ncRNAs for the treatment of other types of cancers, which will be explored in the following section.

In a study on hepatocellular carcinoma HepG2 and Huh-7 cell lines, aspirin has been found to inhibit the proliferation of liver tumors by inhibiting P4HA2 through lncRNA LMCD1-AS1(Bgee database: corpus callosum, sural nerve, testis) -induced sequestering of Let-7g [54, 105]. A bioinformatic study based on samples collected from 40 breast cancer patients, who participated in an Iranian randomized control trial (IRCT2016080818745N11), identified 5 lncRNAs, 12 miRNAs, and 10 genes that were statistically altered by low doses of aspirin. Aspirin appears to induce miR-302/367(Bgee database: miR-302a, placenta, calcaneal tendon and duodenum; miR-367, placenta, adrenal tissue, bone marrow) to stimulate forkhead box D3 (FOXD3), thereby inhibiting breast cancer migration, angiogenesis, and invasion [54, 106]. Genomic analysis has identified lncRNA NEAT1(Bgee database: thyroid gland, nipple, renal medulla) and LOC152578 (Bgee database: placenta, testis, ventricular zone) as key players in aspirin-induced tumor suppression in human colorectal cancer cell lines HCT116, SW620, and DLD1 [54, 107].

Furthermore, the overexpression of miRNAs miR-34a and miR-34b/c(Bgee database: miR-34a in liver, gastrocnemius and adrenal gland ; miR-34b, olfactory segment of nasal mucosa, skin of abdomen, amygdala; miR-34c, testis) has been found to be critical for the antitumor effects of aspirin in p53-deleted HCT116 colorectal cancer cells [54, 108]. In human cholangiocarcinoma HuCCT‑1 tumor cells, aspirin stimulates miR‑340‑5p(Bgee database: miR‑340, calcaneal tendon, adrenal tissue and blood), which hinders the proliferation of HuCCT‑1 cells and suppresses cyclin D1. It is noteworthy that aspirin induces cell cycle arrest at G0/G1 phase, but no evidence of apoptosis was observed [54, 109]. A study involving 6 hepatocellular carcinoma cells lines (HLE, HLF, Huh-7, PLC/PRF/5, Hep-3B, Li-7) and in vivo nude mice model of Huh‑7-xenografted tumors showed that aspirin inhibits tumor growth partially through the miRNA‑137(Bgee database: blood, adrenal gland cortex, vagina)/EGFR pathway, resulting in apoptosis and cell cycle arrest [54, 110]. The proliferation of pancreatic adenocarcinoma cells PANC-1 and PK-8 has been suppressed by aspirin, in part by downregulating GSK-3β. Meanwhile, 274 miRNAs in PANC-1 cells and 30 miRNAs in PK-8 cells were upregulated, and 294 miRNAs in PANC-1 cells and 13 miRNAs in PK-8 cells were suppressed during the process, again highlighting the active role that ncRNAs play in the antitumor effect of aspirin [111]. In a non-small cell lung cancer (NSCLC) A549 cell line, aspirin promoted the expression of miR-135b(Bgee database: olfactory segment of nasal mucosa, islet of Langerhans, blood) and miR-210, leading to G2/M cell cycle arrest under hypoxic conditions and impeding tumor growth [112].

#### Future directions

The success rate of novel cancer drugs passing all three phases clinical trials is very low; it is estimated at only 3.4% for novel therapeutic drugs and 1.2% for orphan drugs. Selecting patient candidates with biomarkers can elevate the success rate to up to 10.7% [113]. Thus, it is unrealistic to expect a sudden influx of novel antitumor therapeutic drugs on the market, offered at reasonable prices with sufficient supply, and available to the general public anytime soon.

Fortunately, the latest developments in machine learning are providing a ray of hope for repurposing traditional drugs for novel antitumor treatments. Computational advancements, combined with machine learning and artificial intelligence (including deep learning) methods, could facilitate the direct simulation of drug-target interactions, identification of novel targets, and optimization of existing drug chemical structures. With more comprehensive biological pathway analysis based on "omics" data, repurposing traditional drugs could become more efficient and convenient, accelerating current research [114].

Innovations in patient-derived xenografts are also noteworthy. Implanting actual patient tumors into humanized mice could improve the accuracy of novel therapeutic drugs and advance personalized medicine [115]. Existing approved traditional drugs could be quickly selected or even combined to treat patients who match specific patterns in their genome, transcriptome, and translatome profiles.

The recent discovery and investigation of RNA-modifying proteins (RMPs) have revealed their critical role in chemical modifications of RNA and have identified them as novel therapeutic targets for cancer [116]. Furthermore, the advancement of CRISPR-based techniques has led to the generation of next-generation RNA editing techniques. Various ADAR-based methods allow for precise RNA editing [117]. Therefore, it is undeniable that more promising treatment plans are being developed by combining traditional therapy with recent advancements in our understanding of RNA and RNA editing techniques.

The overlapping hallmarks between aging and cancer, along with the fact that cancer-related morbidity and mortality rates drop rapidly after the age of 90, have raised questions about the extent to which aging is related to cancer. They also suggest that the biology of aging might provide us with a deeper understanding of cancer pathology as well as potential novel therapeutic targets. This calls for increased collaboration between oncologists and geriatricians [118].

**Fig. 4:**
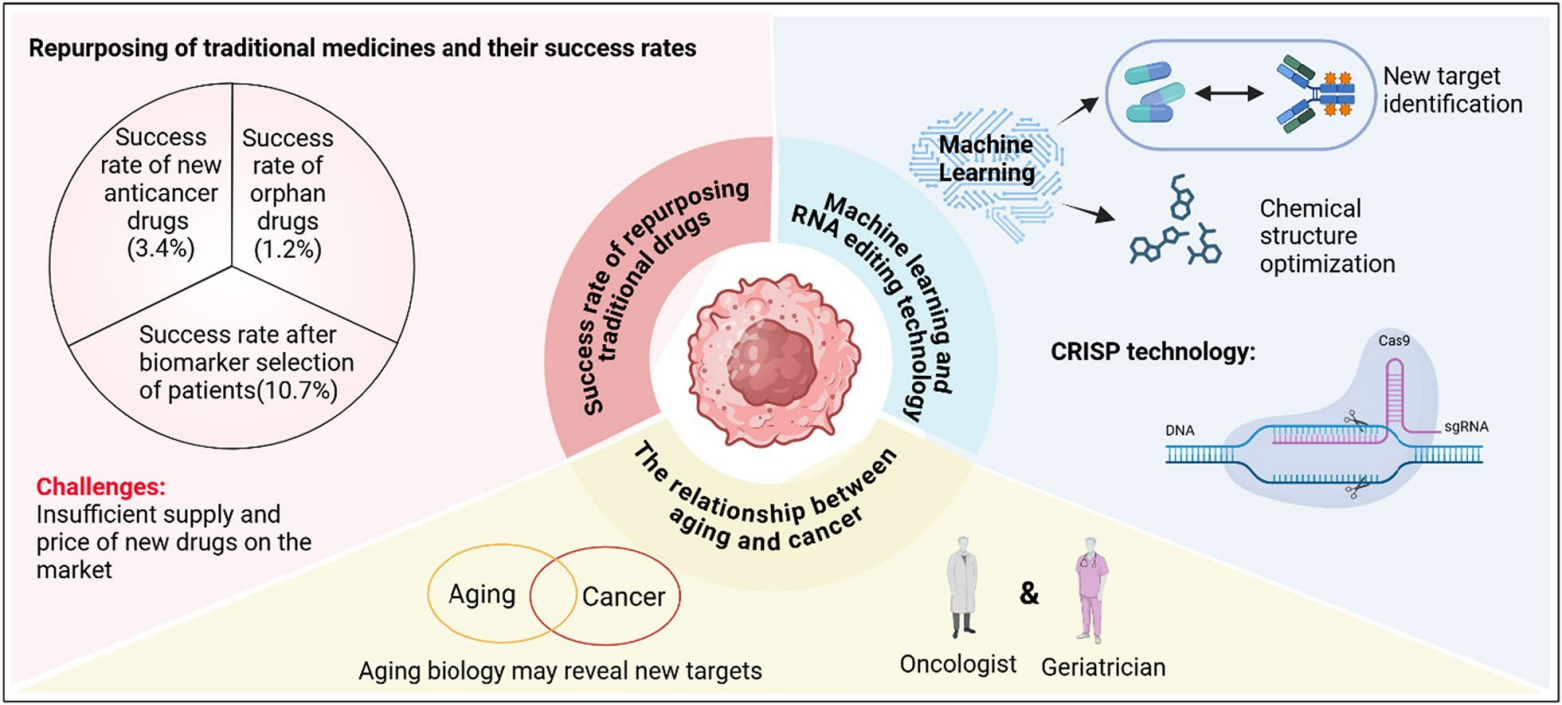
The latest developments and advancements of repurposing traditional drugs against GB. The success rate of traditional drug repurposing is improved by selecting patient biomarkers, among which the success rate of new anticancer drugs is 3.4% and that of orphan drugs is 1.2%. After biomarker screening, the success rate can be increased to 10.7% [113]. At the same time, machine learning plays an important role in drug development, promoting the simulation of drug-target interactions through new target identification and chemical structure optimization, and combining CRISPR technology to achieve precise RNA editing [117]

Overall, in alignment with these latest developments and advancements, adjusting traditional drugs by adjuvant therapy becomes more plausible for treating previously intractable cancers such as GBM, as shown in Fig. 4. The success rate of traditional drug repurposing is improved by selecting patient biomarkers, among which the success rate of new anticancer drugs is 3.4% and that of orphan drugs is 1.2%. After biomarker screening, the success rate can be increased to 10.7% [113]. At the same time, machine learning plays an important role in drug development, promoting the simulation of drug-target interactions through new target identification and chemical structure optimization, and combining CRISPR technology to achieve precise RNA editing [117]. On this basis, aging biology may reveal new therapeutic targets, emphasizing the importance of cooperation between oncologists and geriatricians, in order to develop more effective personalized treatment plans [118].

## Conclusions

Recent advancements in recognizing the functions of ncRNAs have provided researchers with novel therapeutic targets, as well as new tools to adjust traditional drugs for use against GBM. Unlike the development of new anticancer drugs, which is time-consuming and financially intensive with a very low success rate in clinical trials, enhancing efficacy of pre-existing FDA-approved Temozolomide, Disulfiram, and Aspirin by adjuvant therapies can significantly reduce research costs, accelerate the clinical trial process, and offer affordable prices to cancer patients (Table 1 and 2).

**Table 1:** Examples of “old drugs, new tricks”

Drugs	Application (Old)	Application (New)	Mechanism
Aspirin	Antipyretic, analgesic, anti-inflammatory	Cardiovascular disease prevention, cancer prevention	Inhibits inflammation and regulates cell signaling pathway
Metformin	Hypoglycemic	Cardiovascular protection, tumor suppression, anti-aging	Regulate cell metabolism and induce cell apoptosis
Thalidomide	Antiemetic for pregnant women (banned)	Multiple myeloma, leprosy erythema nodosum	Promotes TNF-α mRNA degradation
Lansoprazole	peptic ulcer	Tumor multidrug resistance, obesity and metabolic diseases	Inhibits ABC transporter protein activity and promotes fat thermogenesis
Sildenafil	Cardiovascular disease (unsuccessful)	Erectile dysfunction, pulmonary hypertension	Dilates blood vessels and improves blood flow
Desloratadine	Antiallergic	Anti-liver cancer	Target the NMT1 protein and inhibit its enzyme activity
Dapagliflozin	Diabetes	Heart failure, chronic kidney disease	Targeted ERR alpha-OAT1 axis to promote uric acid excretion and improve renal fibrosis
Arsenic trioxide	Sores	Acute promyelocytic Leukemia (APL)	Induced differentiation and apoptosis of leukemia cells

**Table 2:** ncRNAs as potential GBM therapeutic targets

ncRNAs	Variable	Drugs	Functions in GBM	References
HOTAIR	lncRNA		crucial regulators of occurrence, progression, and drug resistance of GBM	[43, 44]
MALAT1	lncRNA		crucial regulators of occurrence, progression, and drug resistance of GBM	[43, 44]
SBF2-AS1	lncRNA		crucial regulators of occurrence, progression, and drug resistance of GBM	[43, 44]
Mir-21	microRNA		its suppression can trigger anti-apoptotic and pro-survival pathways	[48]
FOXD2-AS1	lncRNA	TMZ	knockdown of FOXD2-AS1 can attenuate TMZ resistance in human glioma cell lines U251 and A172	[53, 54]
UCA1	lncRNA	TMZ	the lncRNA UCA1/miR-182-5p/MGMT regulatory axis can play a pivotal role in reducing TMZ resistance	[54, 55]
lnc-TALC	lncRNA	TMZ	lnc-TALC promotes TMZ resistance from competitively binding to miR-20b-3p and facilitate the expression of c-Met and concurrently upregulate MGMT expression in human GBM	[54, 56]
TP73-AS1	lncRNA	TMZ	responsible to TMZ resistance as well as to mechanisms of brain aging	[54, 61]
ZBED3-AS1	lncRNA	TMZ	downregulation of ZBED3-AS1 leads to the activation of Thrombomodulin	[54, 62]
miR-519a	microRNA	TMZ	induce programmed cell deaths in GBM cells,	[54, 62]
miR-182	microRNA	TMZ	a promoter of differentiation in GBM cell lines, an inhibitor of GBM cell proliferation	[49, 58]
miR-606	microRNA	DFS	regulate the mRNA of the FDX1 to inhibit the glycolysis and proliferation of GBM cells	[54]
LMCD1-AS1	lncRNA	Aspirin	aspirin has been found to inhibit the proliferation of liver tumors by inhibiting P4HA2 through lncRNA LMCD1-AS1(Bgee database: corpus callosum, sural nerve, testis) -induced sequestering of Let-7 g	[54, 105]
miR-302/367	microRNA	Aspirin	Aspirin appears to induce miR-302/367 to stimulate forkhead box D3 (FOXD3), thereby inhibiting breast cancer migration, angiogenesis, and invasion	^[54, 106]^
NEAT1	lncRNA	Aspirin	key players in aspirin-induced tumor suppression in human colorectal cancer cell lines	^[54, 107]^
LOC152578	lncRNA	Aspirin	key players in aspirin-induced tumor suppression in human colorectal cancer cell lines	^[54, 107]^
miR-34a	microRNA	Aspirin	critical for the antitumor effects of aspirin in p53-deleted HCT116 colorectal cancer cells	^[54, 108]^
miR-34b/c	microRNA	Aspirin	critical for the antitumor effects of aspirin in p53-deleted HCT116 colorectal cancer cells	^[54, 108]^

## Declarations

### Availability of data and material

No datasets were generated or analyzed during the current study.
